# More insights into the immunosuppressive potential of tumor exosomes

**DOI:** 10.1186/1479-5876-6-63

**Published:** 2008-10-30

**Authors:** Veronica Huber, Paola Filipazzi, Manuela Iero, Stefano Fais, Licia Rivoltini

**Affiliations:** 1 Unit of Immunotherapy of Human Tumors, Fondazione IRCCS Istituto Nazionale Tumori, Milan, Italy; 2 Department of Drug Research and Evaluation, Anti-Tumor Drugs Section, Istituto Superiore di Sanità, Rome, Italy

We did read with great interest the recent review published by Ichim et al on the potential role of tumor exosomes as immune escape mechanism [1], and we were pleased to see that the authors shared our original idea that these organelles may represent a crucial tool of immunosuppression in cancer [2,3]. Indeed, although tumor cells are well acknowledged to affect immune functions through the release of diverse soluble factors or cell-to-cell contact mediated mechanisms [4,5], the involvement of alternative pathways based on the secretion of membrane microvesicles has been so far largely unappreciated [6]. Exosomes are endosome-derived organelles of 50–100 nm size, actively secreted by virtually all cell types through an exocytosis pathway that is used under normal as well as pathological conditions [6]. Their first description can be attributed to the biochemist Rose Johnstone, who reported in her 1980s investigations about these lipid-encased particles produced as a mechanism for shedding of specific membrane functions during reticulocyte maturation [7]. Since then, these curious microvesicles lingered in obscurity, although several reports kept referring to exosomes as potential pathway utilized by different cell types to eliminate cellular material or establish intercellular cross-talk [8]. Finally in 1996 these microparticles were recognized for their central role in antigen presentation with the work of Graça Raposo and Hans Geuze of Utrecht University in the Netherlands, who reported that exosomes secreted by B cells could promote T cell cross-priming through the expression of HLA/peptide complexes [6]. Based on these and following observations about the role of exosomes in antigen presentation, the exacerbated production of these vesicles by tumor cells was initially welcomed as a process potentially involved in the induction and maintenance of tumor immunity [9]. Indeed, the expression of a large panel of tumor proteins with antigenic properties, like MelanA/Mart-1 and gp100 in melanoma-derived exosomes, and CEA and HER2 in exosomes produced by carcinoma cells [9-11], supported the role of these organelles as cell-free source of tumor antigens for T cell priming and paved the way to clinical trials based on vaccination with tumor exosomes in patients with advanced disease [12].

However, following studies from several groups including ours have progressively suggested that these vesicles, being close replicas of the originating cancer cells, could transport not only antigenic material but also molecules responsible for the detrimental effects exerted by tumor cells on the immune system [6,13,14].

As most researchers, we entered the exosome field by chance, in the course of studies on FasL as tumor immune escape mechanism in human cancer. Indeed, despite the first report on the expression of FasL by melanoma [15], we could not succeed in detecting stable membrane expression of this pro-apoptotic molecule on such tumor cells. However, by using immunocytochemistry and immunoelectron microscopy, we found that FasL was indeed detectable intracellularly, as localized in defined endocytic compartments with a clear secretory behaviour. Thanks to this initial observation, we discovered that human melanoma as well as colon carcinoma cells constitutively release FasL and TRAIL-expressing exosomes, which induce death by apoptosis in activated T cells [10,11]. This evidence, confirmed also by Whiteside and coworkers in head and neck cancer [16], highlights a germane role of microvesicular structures in counteracting tumor immunity by simply eliminating activated T cells bearing tumor-reactive TCR. This might occur even at distance (in peripheral lymphoid organs, bone marrow, peripheral blood, and biological fluids) without the need for a direct cell-to-cell contact. And given the evidence that exosome of probable tumor origin are abundantly found in plasma or pathological effusions of cancer patients [9,11], it can be easily hypothesized that this pathway may contribute to the in vivo moulding of immune as well as other cancer-related host responses. More recent studies have then reported that the detrimental effect of tumor exosome on immune effector functions is not restricted to T cells but can target NK cells as well, through the skewing of IL-2 responsiveness in favour of regulatory T cells [17] or down-modulation of NKG2D expression [18]. Moreover, the negative influence of tumor exosomes on specific immunity goes beyond T and NK cells and may also target crucial up-stream steps for T cell cross-priming, namely dendritic cell (DC) differentiation. In fact, we have more recently observed that the presence of tumor exosomes during monocyte differentiation into DC skews the whole process toward the generation of aberrant cells expressing myeloid markers (such as CD14 and CD11b), lacking or bearing low levels of co-stimulatory molecules (like HLA-DR, CD80 and CD86) and spontaneously secreting TGF-beta [19,20]. These cells, which exert a strong immunosuppressive activity on T cell proliferation and function, highly resemble the "myeloid-derived suppressor cell" subset described to accumulate with tumor progression in different murine models [21]. Interestingly enough, melanoma patients with advanced disease have high levels of these CD14+ HLA-DR neg/low TGF beta-secreting cells in their peripheral blood, and this frequency appears to be a disadvantageous factor for the development of immune responses to tumor vaccines [20]. These findings, which again were confirmed in other experimental settings [22], define a very sharp profile of tumor exosomes as efficient delivery system of immunosuppression, contributing to the maintenance of an immune tolerance state in cancer bearing hosts.

The interest on exosomes has recently spread out as these vesicles are being found involved in a wide spectrum of physiological and pathological cellular events, as alternative tools of intercellular communication and paracrine functions [23], or as pathogenic pathways in viral [24] and prion-related diseases [25]. Thanks to their peculiar lipid composition, highly enriched in ceramide [26], sphingomyelin, cholesterol and GM3 glycolipid [27], exosomes may serve as a more advantageous carrier of signal delivery favouring stable conformational conditions, increased bioactivity, improved bio-distribution and amplified target interaction of their protein content with respect to soluble molecules. In the last years, literature is indeed flourishing with examples proving the role of tumor exosomes in the transfer of growth factors and cognate receptors to homologous or heterologous target cells. For instance glioma cells can share EGFR by intercellular transfer of membrane-derived microvesicles ('oncosomes') [28], or pancreatic carcinoma can deliver exosomes overexpressing tetraspanin family members and promoting autocrine secretion of MMP and VEGF [29]. The evidence that these organelles can also shape protein synthesis through the transfer of functional mRNAs and microRNAs, as recently reported in transformed mastocytes [30], adds then a further pathway to the potential modulating properties of these peculiar organelles.

If tumor exosomes are such a powerful instrument of environmental shaping, then getting rid of them should significantly affect cancer cell ability to survive and expand in vivo. In their review, Ichim et al propose a physical approach based on the extracorporeal removal of exosomes from plasma of cancer patients, through a novel hollow-fiber cartridge (Hemopurifier™) designed to eliminate particles expressing heavily glycosylated surface proteins, like in case of viruses and cancer microvesicles [1]. The approach could be further implemented by the attachment of clinical grade molecules and antibodies to the cartridge resin, to allow microvesicle depletion on the basis of selected marker expression. Although interesting, feasible and potentially effective in the short-term, this strategy could only have an impact on circulating exosomes, leaving vesicles accumulating at tumor tissue level, in draining lymph nodes or in other relevant lymphoid compartments, still available for immunosuppressive functions. Obviously, physical removal would not interfere with the process of exosome secretion, and would indiscriminately eliminate vesicles from both pathological and normal cells. In alternative, we are considering to intervene on tumor exosome secretion by inhibiting up-stream crucial pathways involved in the process. Although definitive information on the mechanisms regulating microvesicle release by cancer cells are presently scantly, preliminary data suggest that particular molecules, such as drugs interfering with microtubule stability (taxanes and vinca alkaloids) [M. Iero, unpublished observations] or additional microtubule-disturbing molecules like vincristine [31], can affect endosomal stability and reduce microvesicle release. Similarly, drugs targeting the activity of enzymatic efflux pumps expressed on acidic vacuoles, such as vacuolar-ATPases inhibitors, could selectively alter exosome trafficking and release in tumor cells [Iero et al., unpublished, [32]]. Benefits from modulation of exosome secretion could also come from qualitatively shaping protein composition of secreted microvesicles with drugs altering biological features of tumor vesicles, such in the case of curcumin, a natural polyphenol which has been shown to reduce immunosuppressive functions of breast carcinoma-secreted exosomes [33].

A more specific approach would be instead to identify the molecular mechanisms responsible for the immunosuppressive activity and the microenvironment remodelling effects of tumor exosomes [34], to selectively interfere with these pathways through specific antibodies, antisense oligonucleotides or signalling inhibitors.

Independently from the tool utilized for diminishing exosome release by tumor cells, the most challenging task of the near future is to prove that interfering with microvesicle secretion in vivo may indeed result in tumor growth arrest or slow-down thanks to the recovery of specific immunity and the interruption of paracrine/autocrine loops in tumor microenvironment. Prior to any clinical intervention, experimental studies in animal models should thus be performed to assess what is the real impact that these vesicles play in cancer progression and what is the expected benefit of shutting off their production at tumor site.

## Authors contributions

VH  was responsible for editorial writing, senior scientist responsible for the studies on the immunosuppressive functions of tumor exosomes.    PF was responsible for editorial reviewing, scientist responsible for the studies on the induction of myeloid-derived suppressor cells by tumor exosomes.    MI was responsible for editorial reviewing, scientist responsible for the studies on the modulation of exosome release by tumor   cells.    SF  was responsible for editorial reviewing, external collaborator in the studies on the involvement of proton-pump inhibitors on exosome release.    LR  was responsible for editorial writing and reviewing, supervisor of the  studies on tumor exosomes
